# A new species of *Litarachna* (Acari, Hydrachnidia, Pontarachnidae) from a Caribbean mesophotic coral ecosystem

**DOI:** 10.3897/zookeys.425.8110

**Published:** 2014-07-15

**Authors:** Vladimir Pešić, Tapas Chatterjee, Monica Alfaro, Nikolaos V. Schizas

**Affiliations:** 1 Department of Biology, University of Montenegro, Cetinjski put b.b., 81000 Podgorica, Montenegro; 2 Department of Biology, Indian School of Learning, I.S.M. Annexe, P.O. – I.S.M., Dhanbad-826004, Jharkhand, India; 3 Department of Biology, University of Puerto Rico, Mayagüez, Call Box 9000, Mayagüez, PR 00681, USA; 4 Department of Marine Sciences, University of Puerto Rico, Mayagüez, Call Box 9000, Mayagüez, PR 00681, USA

**Keywords:** Water mites, taxonomy, marine environment, Puerto Rico, biodiversity

## Abstract

New records of pontarachnid mites (Acari: Hydrachnidia) from the Caribbean island of Puerto Rico are presented. *Litarachna lopezae* **sp. n.**, from substrata collected from Bajo de Sico, a mesophotic coral reef ecosystem in Mona Passage off Puerto Rico, is described as new to science. The new species was collected from nearly 70 m depth, the greatest depth from which pontarachnid mites have been found until now. In addition, a *Litarachna* sp. was also found in association with the tube of the polychaete *Sabellastarte magnifica* (Shaw, 1800) at the shallow waters of north Puerto Rico.

## Introduction

The water mite family Pontarachnidae Koenike, 1910, the only family of the Hydrachnidia occurring in the marine environment, represents a well-defined monophyletic clade. Most species are reported from the littoral zone of marine waters in tropical and subtropical areas. In Australia and South Africa species have been recorded from estuarine freshwaters, and two species in South Korea are known to live only in marine interstitial environments (Pešić 2013). Nothing is known about the life cycle of the Pontarachnidae. So far, three species are known from the Caribbean Sea, i.e. *Litarachna degiustii* Cook, 1958 (Bimini, Bahamas – Cook 1958, Netherlands Antilles – Pešić et al. 2008), *Litarachna caribica* Pešić, Chatterjee & Schizas, 2008 (Netherlands Antilles – Pešić et al. 2008) and *Pontarachna nemethi* Pešić, Chatterjee & Schizas, 2012 (Vieques Island of Puerto Rico – Pešić et al. 2012b).

Mesophotic coral ecosystems (MCEs) are light-dependent habitats dominated by macroalgae, sponges and scleractinian corals and are found on the insular and continental slopes of Caribbean islands between 30 and 100 m (Locker et al. 2010). Even at the lower depth range (70–100 m), there is enough light for photosynthesis to take place enhancing the growth of several scleractinian coral species (e.g. *Agaricia* spp., *Montastraea* spp.) and algae. The MCEs of Puerto Rico represent a potential biodiversity hotspot for marine arthropods and so far 2 mites (Pešić et al. 2012b, current paper), 1 harpacticoid copepod (Corgosinho and Schizas 2013) and 9 cumaceans (Petrescu et al. 2012, 2013, 2014a, b) new to science have been described.

In this paper we describe a new species, *Litarachna lopezae* sp. n. collected during the second of 3 mesophotic cruises (2010–2012) organized by the University of Puerto Rico at Mayagüez (UPRM), the Caribbean Coral Reef Institute (CCRI) and the Department of Marine Sciences (DMS) of UPRM (Sherman et al. 2013). We also describe the female specimen of a tentative new species which was found in association with the polychaete *Sabellastarte magnifica* (Shaw, 1800) from a shallow water habitat in north Puerto Rico.

## Materials and methods

Material examined in the present study was collected from Bajo de Sico (18°14'41.676"N, 67°24'45.791"W), a mesophotic reef formation located in Mona Passage off Puerto Rico. During the 2011 Mesophotic Cruise of DMS-UPRM, divers equipped with Tri-Mix Rebreathers collected substrata (loose rubble, corals, sponges, algae) from 69.5 m depth and placed them in sealed plastic bags. As soon as the samples returned to the surface they were placed on a 1 mm and 0.125 mm sieves. Samples were washed with filtered seawater and the portion of fauna retained on the 0.125 mm sieve was preserved in 95% ethanol. One specimen was collected from Rio Grande, Puerto Rico (18°25'11.86"N, 65°47'40.43"W) from marine littoral. This specimen was found while tubes of the polychaete *Sabellastarte magnifica* were washed into a 0.063 mm sieve. Mites and other fauna were extracted by Alexandra Galindo and the fourth author with the aid of a stereomicroscope and placed back in 95% ethanol. Slide-mounting was done in Hoyer's fluid and water mites were treated in laboratory as decribed by Gerecke et al. (2007). All drawings were prepared using a drawing tube attached to a Olympus BX43 brightfield microscope. The holotype and paratypes are planned to be deposited in the Museum of Natural History of Montenegro in Podgorica.

All measurements are given in µm. The following abbreviations are used: Cx-I = first coxae, dL = dorsal length, H = height, L = length, I/II/III/IV-L-1-6 = first to sixth segments of the first to fourth legs, P-1 to P-5 = palp segments 1 to 5, vL = ventral length, W = width.

## Systematics

### Genus *Litarachna* Walter, 1925

#### Litarachna lopezae sp. n.

Taxon classificationAnimaliaTrombidiformesPontarachnidae

http://zoobank.org/4C577A5A-9287-476A-A611-12DD3667687C

**Figure 1.**
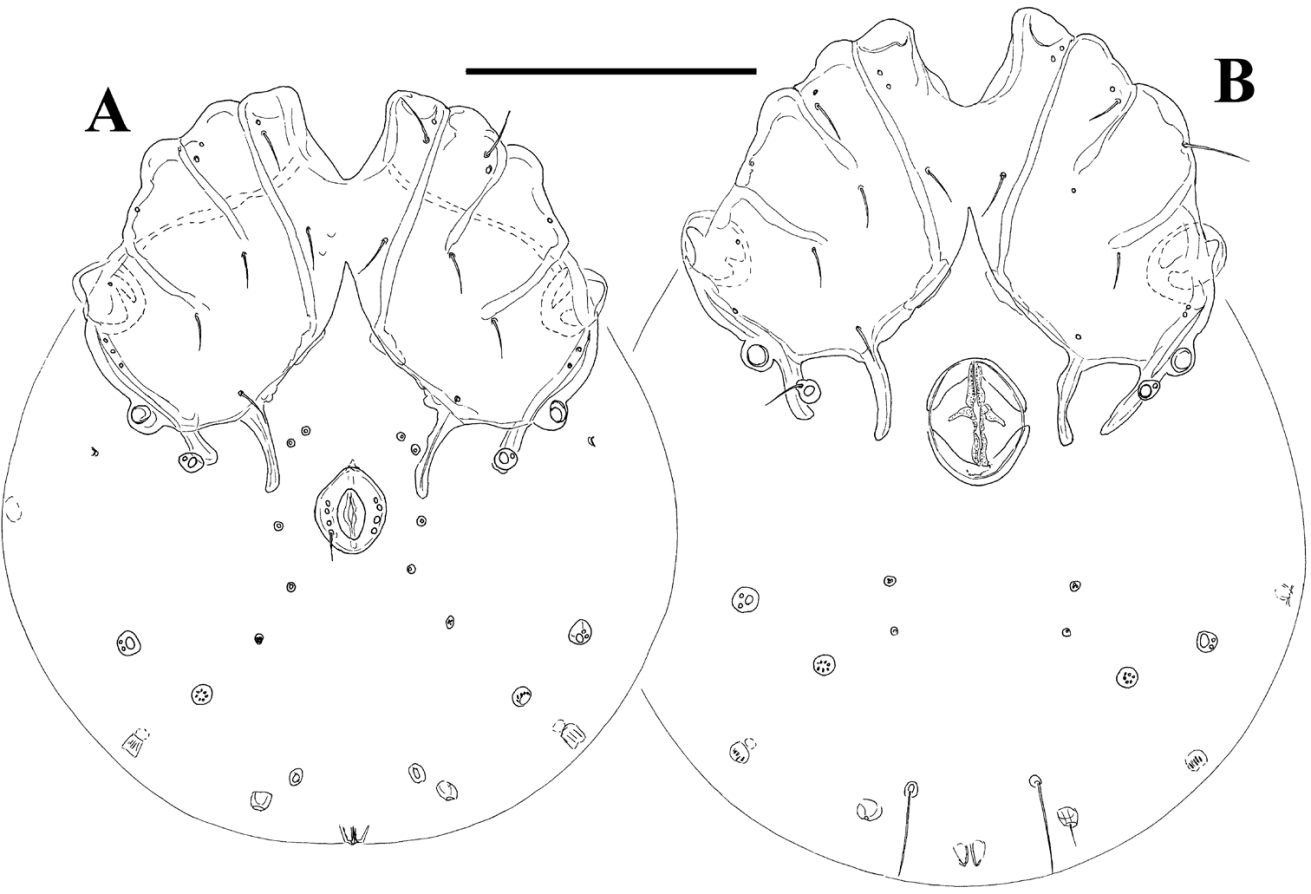
*Litarachna lopezae* sp. n., Bajo de Sico (**A** male, **B** female): idiosoma, ventral view. Scale bar = 100 µm.

**Figure 2.**
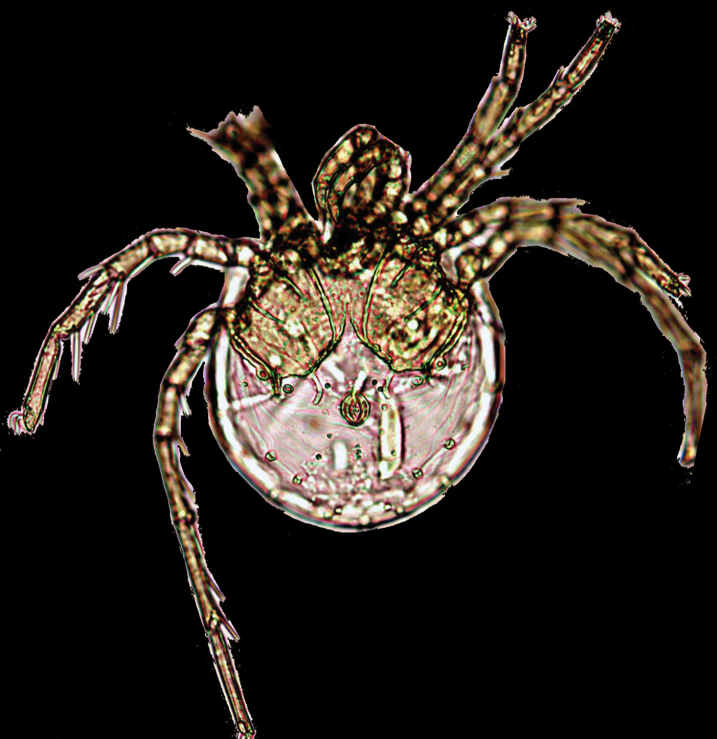
Photograph of *Litarachna lopezae* sp. n., male, Bajo de Sico: ventral view.

**Figure 3.**
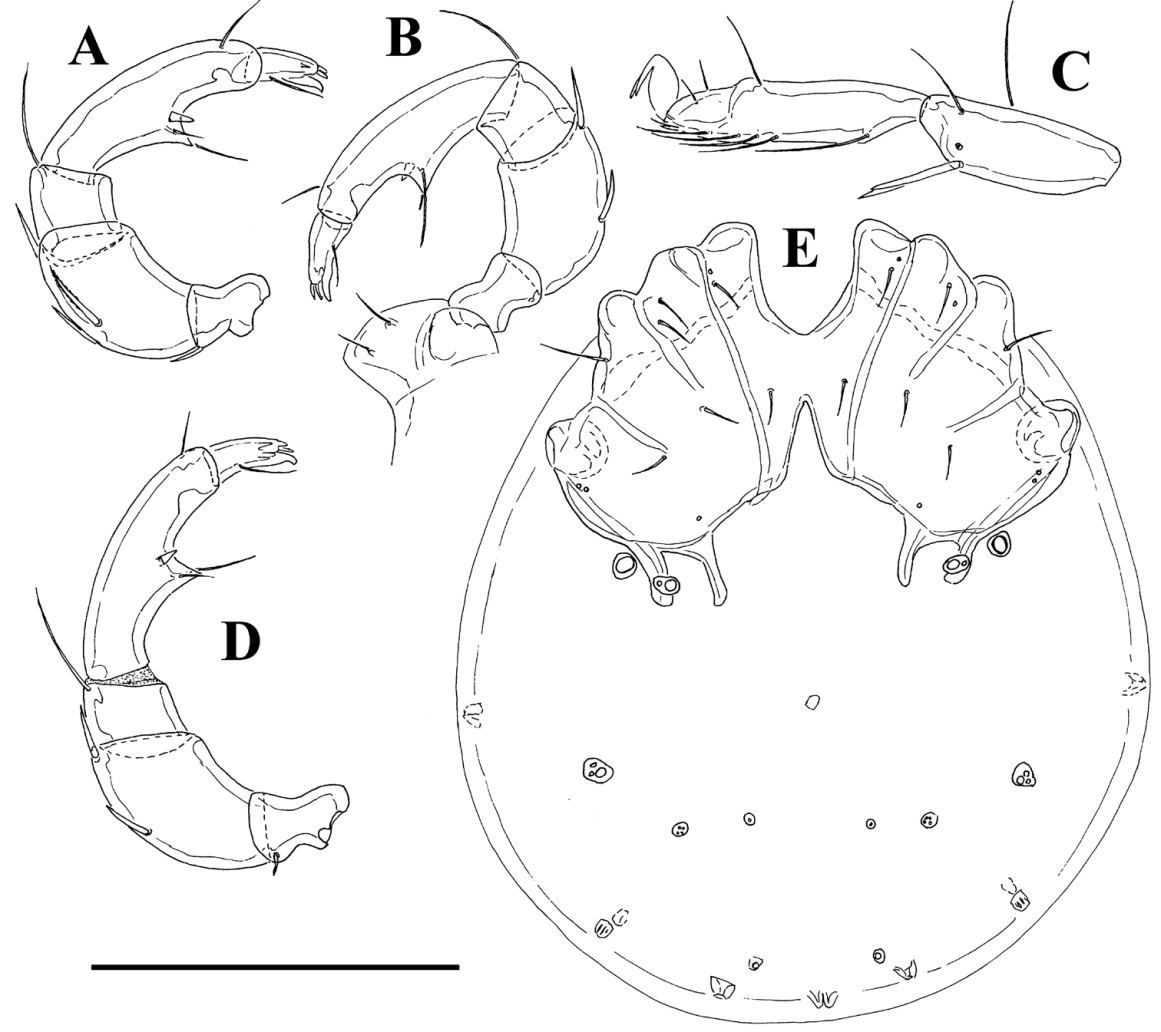
*Litarachna lopezae* sp. n., Bajo de Sico (A-C = male, D = female, E = deutonymph): **A–B, D** palp **C** I-L-5-6 **E** idiosoma, ventral view. Scale bar = 100 µm.

Figs 1A–B , 2 , 3A–E

##### Type series.

Holotype male, dissected and slide mounted, Puerto Rico, Bajo de Sico, 18°14'41.676"N, 67°24'45.791"W, depth 69.5 m, 20.iv.2011. Paratypes: three males, two females, one deutonymph, same data as holotype, one male and one female dissected and slide mounted.

##### Diagnosis.

Adults. Idiosoma small (L 250-300 µm); first coxal plates fused; glandularium-like structure fused with Cx-IV, a pair of small platelets with (according to Wiles et al. 2002) coxoglandularia 4 and associated setae free in the integument near the lateral posterior apodemes of Cx-IV; ventral margin of P-4 with a setal tubercle and a small peg-like seta.

##### Description.

*General features* – First coxal plates fused medially; suture lines Cx-I/II complete, suture line Cx-II/III and Cx-III/IV incomplete. Posterior margin of Cx-IV with two pairs of apodemes of moderate length, the medial longer than lateral ones, extending beyond anterior margin of genital field; glandularium-like structure on the outer side of lateral posterior apodemes of Cx-IV, fused with the fourth coxal plates; a pair of small platelets with coxoglandularia 4 and associated setae free in the integument near the lateral posterior apodemes of Cx-IV; posterior to the genital field a pair of platelets with three pores, and three pairs of small wheel-like acetabula, with relatively few radiating spokes. Excretory pore unsclerotized, near posterior end of idiosoma. Palp: ventral margin of P-2 concave without extension; ventral margin of P-4 with a setal tubercle and a small peg-like seta. Legs (Fig. 3C): swimming seta numbers: III-L-5, 1; IV-L-4, 1; IV-L-5, 1. Male: genital field consisting of a sclerotized ring with four pairs of setae; four pairs of perigenital setae free in integument around genital field. Female: pre and postgenital sclerites bowed.

Deutonymph. As in adults but lacking genital field; glandularium-like structure free in the integument on the outer side of lateral posterior apodemes of Cx-IV.

*Measurements.* Male (holotype, in parentheses paratype, n = 1) – Idiosoma (ventral view, Figs 1A, 2) L 258 (268), W 234 (230); coxal field L 116 (106), Cx-III W 154 (158); ring-shaped genital plate L 29 (31), W 25 (24); chelicera total L (116). Palp (Figs 3A-B): total L 183 (182), dL/H, L/H ratio: P-1, 17/12, 1.38 (16/12, 1.3); P-2, 52/28, 1.89 (53/29, 1.86); P-3, 22/23, 0.97 (23/23, 1.0); P-4, 67/17, 4.0 (67/17, 4.0); P-5, 25/10, 2.5 (23/10. 2.4); dL P-2/P-4 ratio 0.78 (0.79); dL of I-L-3-6: 35, 34, 53 (52), 71 (73); I-L-6 H 17 (15), I-L-6 dL/H ratio 4.3 (4.7); dL of IV-L-2-6: 35, 42, 68, 88, 88.

Female – Idiosoma (ventral view, Fig. 1B) L 295, W 237; coxal field L 118, Cx-III W 160; genital field L 44, pregenital sclerite W 34, postgenital sclerite W 35; chelicera total L 134. Palp (Fig. 3D): total L 190, dL/H, L/H ratio: P-1, 16/12, 1.3; P-2, 59/31, 1.9; P-3, 19/24, 0.78; P-4, 71/17, 4.2; P-5, 25/11, 2.35; dL P-2/P-4 ratio 0.83; dL of IV-L-4-6: 74, 91, 92.

Deutonymph – Idiosoma (ventral view, Fig. 3E) L 220, W 190; coxal field L 90, Cx-III W 119; palp: total L 129, dL/H, L/H ratio: P-1, 13/9, 1.4; P-2, 34/20, 1.7; P-3, 17/17, 1.0; P-4, 48/13, 3.7; P-5, 17/7, 2.46 dL P-2/P-4 ratio 0.71.

##### Etymology.

This species is named after the famous Puerto Rican singer Jennifer Lopez.

##### Remarks.

Six *Litarachna* species have their first coxal plates fused, i.e., *Litarachna degiustii* Cook, 1958 (Caribbean Sea – Cook 1958, Pešić et al. 2008), *Litarachna amnicola* Cook, 1986 (Tasmania – Cook 1986, Pešić and Smit 2009), *Litarachna brasiliensis* Smit, 2007 (Brazil – Smit 2007), *Litarachna caribica* Pešić, Chatterjee & Schizas, 2008 (Caribbean Sea – Pešić et al. 2008), *Litarachna indica* Pešić, Chatterjee & Ingole, 2012 (West Indian coast – Pešić et al. 2012a) and *Litarachna minuta* Pešić, Chatterjee & Marshall, 2013 (Brunei Darussalam – Pešić et al. 2013).

Due to the glandularium-like structure fused with posterior margin of Cx-IV, *Litarachna lopezae* sp. n. most closely resembles to *Litarachna minuta*, a species known from a single female from Brunei Bay, but differs by a pair of small platelets with coxoglandularia 4 and associated setae lying free in the integument, not fused with Cx-IV (fused in *Litarachna minuta*).

Moreover, peg-like seta at the base of P-4 ventral projection separates new species from *Litarachna minuta* and other species with fused first coxal plates.

##### Habitat.

The mites were collected from 69.5 m depth. The greatest depth at which pontarachnid mites have been recorded previously was reported by Pešić et al. (2012b) who found *Pontarachna nemethi* in a mesophotic coral ecosystem near Vieques Island of Puerto Rico at 67 m depth.

##### Distribution.

Only known from the type locality.

#### *Litarachna* sp.

Fig. 4A–D

**Material examined.** Puerto Rico, Rio Grande, 18°25'11.86"N, 65°47'40.43"W, depth 0.5 m, 15.ii.2014, one female, dissected and slide mounted.

**Figure 4.**
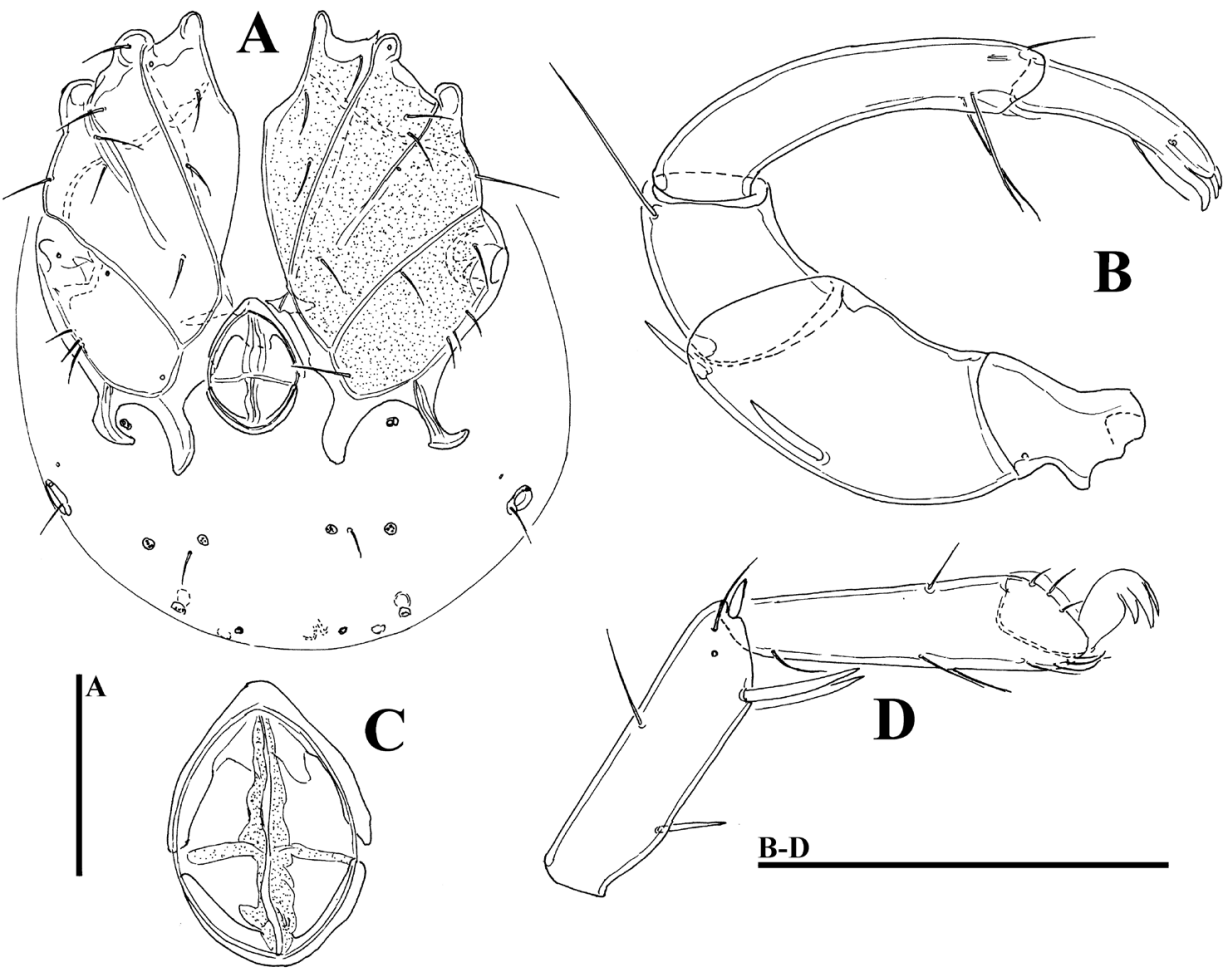
*Litarachna* sp., female, Rio Grande: **A** idiosoma, ventral view **B** palp **C** genital field **D** I-L-5 and -6. Scale bars = 100 µm.

**Description.** Female. *General features* – Cx-I separated medially; suture lines Cx-I/II and Cx-III/IV complete, suture line Cx-II/III incomplete; posterior margin of Cx-IV with two pairs of apodemes of moderate length, the medial broad and longer than lateral ones, extending beyond posterior margin of genital field; pair of small platelets with coxoglandularia 4 and associated setae free in the integument between the posterior apodemes of Cx-IV; pre- and post-genital sclerites strongly bowed, almost touching each other, pregenital sclerite arrow-shaped (Fig. 4C); posterior to the genital field a pair of platelets with three pores and a glandularium-like structure, and three pairs of small wheel-like acetabula, with relatively few radiating spokes; excretory pore unsclerotized, near posterior end of idiosoma. Palp: P-2 ventral margin concave, P-5 longer than 1/2 of P-4. Legs (Fig. 4D): swimming seta numbers: IV-L-4, 1; IV-L-5, 1.

*Measurements* – Idiosoma (ventral view, Fig. 4A) L 323, W 269; coxal field L 197, Cx-III W 219; genital field L 71, pregenital sclerite W 50, postgenital sclerite W 46; chelicera total L. Palp (Fig. 4B): total L 307, dL/H, L/H ratio: P-1, 18/19, 0.97; P-2, 87/44, 1.98; P-3, 46/34, 1-36; P-4, 100/24, 4.1; P-5, 56/14, 4.0, dL P-2/P-4 ratio 0.87; dL of I-L-2-6: 42, 48, 55, 81, 90; I-L-6 H 23, I-L-6 dL/H ratio 3.9; dL of IV-L-2-6: 49, 58, 91, 106, 108.

**Remarks.** The single female from Puerto Rico closely resembles *Litarachna communis* Walter, 1925, a species widespread in Mediterranean (Pešić et al. 2012a), but clearly differs in having more bowed, arrow-shaped pregenital sclerite. Most probably we are dealing with an undescribed species, but since male specimens were not available, a final decision cannot be made.

**Habitat.** The single specimen was collected from a tube of a live *Sabellastarte magnifica* (Shaw, 1800), a large, tubiforous shallow water polychaete of the Caribbean. The polychaete specimens were collected together with their tubes and were washed in a 0.063 mm sieve to examine the associated fauna. Our sampling strategy limits our conclusions whether the female mite was collected from inside or outside the polychaete tube.

## Supplementary Material

XML Treatment for Litarachna lopezae
